# Consumer Demand for Online Dizziness Information: If You Build it, They may Come

**DOI:** 10.3389/fneur.2014.00050

**Published:** 2014-04-16

**Authors:** Kevin A. Kerber, Lesli E. Skolarus, Brian C. Callaghan, Kai Zheng, Yuhao Zhang, Lawrence C. An, James F. Burke

**Affiliations:** ^1^Department of Neurology, University of Michigan Health System, Ann Arbor, MI, USA; ^2^Department of Health and Management Policy, School of Public Health, University of Michigan, Ann Arbor, MI, USA; ^3^School of Information, University of Michigan, Ann Arbor, MI, USA; ^4^Department of Electronic Engineering, Tsinghua University, Beijing, China; ^5^Center for Health Communications Research, University of Michigan, Ann Arbor, MI, USA; ^6^Department of Internal Medicine, University of Michigan Health System, Ann Arbor, MI, USA; ^7^VA Center for Clinical Management and Research, Ann Arbor, MI, USA

**Keywords:** dizziness, vertigo, consumer demand, internet searches

## Abstract

**Objective:** Dizziness is a common reason patients present to doctors, but effective diagnostic tests and treatments for dizziness are underused. The internet is a way to disseminate medical information and is emerging as an intervention platform. The objective of this study was to describe internet searches for dizziness terms to assess the possible consumer demand for internet-based dizziness diagnostic and treatment tools.

**Study Design/Methods:** *Google AdWords* and *Google Insights for Search* were used for keyword search data on the following generic terms: vertigo, dizzy, dizziness, lightheaded, and lightheadedness. Data collected included keyword ideas (i.e., additional keywords identified by Google as being related search terms), global and US only monthly search frequencies, as well as trends in top searches related to dizziness terms from 2004 to 2012. Keywords suggestive of benign paroxysmal positional vertigo (BPPV) or BPPV processes were identified.

**Results:** Of the five generic dizziness terms, vertigo had the most global searches per month (1.83 million) and lightheadedness had the least (90,500). Four BPPV-specific terms had more than 100,000 global searches per month. Three BPPV terms (“positional vertigo,” “benign vertigo,” and “benign positional vertigo”) have been in the list of top searches related to vertigo every quarter since 2004.

**Conclusion:** Substantial demand exists for dizziness information via the internet. Future studies should seek to better characterize the population seeking this information. The magnitude of this potential demand suggests that validated and tested diagnostic and treatment tools could contribute to healthcare efficiencies and patient outcomes.

## Introduction

Dizziness (moderate or severe in intensity) has a lifetime prevalence of up to 30% of the general public and is one of the most common reasons for a physician visit (1–4). Another place where patients are increasingly seeking medical information is the internet (5). In fact, 59% of US adults use the internet to look for health information, typically regarding a specific disease or medical problem (5).

Medical information regarding vestibular topics is found on general internet sources such as academic department websites and also on YouTube (6, 7). Posted comments on the YouTube websites indicate that both patients and providers are seeking out diagnostic and treatment information regarding dizziness (6). For most of these benign paroxysmal positional vertigo (BPPV)-specific websites, the accuracy of the content was determined to be acceptable (6). However, the content of websites that more broadly address dizziness has not been studied and any efforts to create internet-based interventions for dizziness will undoubtedly face challenges and ethical concerns. It is encouraging that prior internet-based interventions have been effective regarding various other health-related topics (8). Furthermore, 10-fold more people report being helped as opposed to being harmed by using medical information found on the internet (5).

To better understand the potential consumer demand for dizziness resources via the internet, we aimed to describe internet searches on dizziness and vertigo. If the demand for internet information regarding dizziness is high, then web-based tools may be able to reach a meaningful proportion of this patient population creating an opportunity to optimize the efficiency and effectiveness of care. This type of data is also necessary for informing the design of future studies, specifically regarding sample size calculations, keyword usage, and more focused clinical topics. We were particularly interested in describing keyword searches related to BPPV, because this disorder has an established treatment which is underused in routine care settings (9–15). The diagnosis and treatment of BPPV may be conducive to dissemination via the internet since prior studies demonstrate that patients can successfully self-treat BPPV if provided with adequate information (16–18).

## Materials and Methods

Two Google internet information sources were used to collect data for this study, *Google AdWords* (using the *Keyword Tool*) (19) and *Google Insights for Search* (subsequently merged into *Google Trends*) (20, 21). From these information sources, data can be obtained regarding actual prior searches on keywords of interest. In this study, we entered the following generic keywords into these programs: vertigo, dizzy, dizziness, lightheaded, and lightheadedness. From *Google AdWords*, data were obtained regarding the number of searches (provided as the approximate number of monthly searches over the prior 12 months) that occurred globally (all searches regardless of the country from which they originated) and limited to searches that originated from the United States.

We also used *Google AdWords* to obtain data on “keyword ideas.” “Keyword ideas” is the Google label for a list of other keywords (and their associated search volumes) determined by Google algorithms to be related to the keyword that the user last entered. We used a two-stage snowball approach to identify the “keyword ideas.” For the main *Google AdWords* searches, the “related” filter was used which restricts the keyword ideas to those terms that contain the keyword that was searched. Additional keyword ideas regarding BPPV or BPPV processes were sought by performing searches on dizziness terms without selecting the related filter because common BPPV search terms may not include a dizziness term (e.g., Epley, BPPV). From this additional search, we found large volumes of searches on “Epley” and “BPPV”; therefore, we also performed separate *Google AdWords* searches on these terms to identify other BPPV-related keyword ideas and search volumes. Search results from the five generic dizziness terms and the BPPV terms were combined and duplicate results excluded. For comparison purposes, we also performed separate searches using other common symptoms (e.g., pain, back pain, headache, weakness, abdominal pain, chest pain) (22) and other neuro-otologic symptoms (i.e., hearing loss and ear pain).

From *Google Insights for Search*, we obtained data regarding trends in our generic keyword search terms (e.g., vertigo, dizzy, dizziness, lightheaded, and lightheadedness) over time which is available from its “top searches” list. The “top searches” list provides a ranking of other terms related to the keyword of interest (in this case, one of the generic dizziness terms) dating back to 2004. The “top searches” list is defined by Google as the most common keywords that were searched by users either before or after the keyword of interest was searched. The data provided include the rank of each of the top searches and a normalized scale value that enables a comparison of the search volumes. The scaled value is obtained by dividing the volume of each keyword in the top searches list by the volume of the highest term from the list during the associated time period, and then multiplying it by 100.

Data were collected from *Google AdWords* searches on September 7, 2012, and from *Google Insights for Search* on August 23, 2012. Descriptive statistics were used to summarize the data using Stata 12.0 (College Park, TX, USA). Overall ranking in *Google Insights for Search* was determined by calculating the median ranking for all terms that were present in at least 50% of the quarters.

## Results

Search volumes for each keyword are presented in Table 1, both at the global level (all searches regardless of country of origin) and also limited to searches originating from the United States. The global searches per month for the five generic dizziness terms was highest for “vertigo” (1.83 million), “dizzy” (1.50 million), and “dizziness” (1.22 million) (Table 1). Compared with these dizziness terms, the global searches per month for other common symptoms were higher for “pain” (37.0 million), “back pain” (2.70 million), and “headache” (2.70 million), but comparable or less for “weakness” (1.50 million), “abdominal pain” (1.20 million), “chest pain” (823,000), and other common symptoms (Table 1).

**Table 1:** **Data regarding approximate volumes of searches on generic dizziness keywords and other symptom comparison terms**.

Keyword	Searches per month (global)	Searches per month (US only)
**DIZZINESS TERMS**		
Vertigo	1,830,000	550,000
Dizzy	1,500,000	550,000
Dizziness	1,220,000	550,000
Lightheaded	201,000	110,000
Lightheadedness	90,500	60,500
**COMPARISON TERMS**		
Pain	37,200,000	13,600,000
Back pain	2,740,000	1,500,000
Headache	2,740,000	1,500,000
Weakness	1,500,000	550,000
Abdominal pain	1,220,000	823,000
Chest pain	823,000	450,000
Numbness	823,000	450,000
Knee pain	673,000	450,000
Hearing loss	450,000	246,000
Ear pain	246,000	165,000

*Google AdWords* provided 1,586 unique keyword ideas related to the dizziness terms. *Google Insights for Search* provided the top searches related to “vertigo,” “dizziness,” and “dizzy” each quarter from 2004 to 2012, and for “lightheaded” and “lightheadedness” from 2007 to 2012 (Table 3 and Table S1 in Supplementary Material). The content of many of these additionally identified terms suggest that the associated searches were for medical purposes (Table 2). Examples of terms that suggest medical searches include “positional vertigo,” “benign vertigo,” “bpv vertigo,” and “what causes dizziness” (Table 2).

**Table 2:** **Examples of keyword ideas and associated approximate volumes of searches provided by AdWords (examples from a total of 1,586 keyword ideas)**.

	Searches per month (global)	Searches per month (US only)
**LIKELY MEDICAL SEARCH TERMS**		
Dizzy vertigo	368,000	110,000
Cause of dizziness	110,000	60,500
Cause of vertigo	74,000	49,500
I feel dizzy	60,500	33,100
Dizziness and nausea	49,500	33,100
Dizziness with headache	40,500	22,200
Treatment of vertigo	40,500	22,200
Dizziness in pregnancy	22,200	12,100
Migraine and vertigo	6,600	3,600
Meclizine for vertigo	1,600	1,300
**BPPV-SPECIFIC EXAMPLES**		
Positional vertigo	201,000	27,100
Benign vertigo	165,000	27,100
Benign positional vertigo	165,000	22,200
BPV vertigo	135,000	4,400
Epley maneuver	40,500	27,100
**LIKELY NON-MEDICAL SEARCH TERMS**		
Vertigo the movie	18,100	5,400
Vertigo Hitchcock	14,800	4,400
Vertigo lyrics	14,800	2,400
Hotel vertigo	9,900	3,600
Vertigo bar	6,600	720
**UNCLASSIFIED SEARCH TERMS**		
Definition of vertigo	9,900	4,400
Vertigo Wikipedia	5,400	1,300
Vertigo hat	140	73
Google vertigo	140	36
Lightheaded hip hop	58	28

**Table 3:** **Top searches related to the search term vertigo, presented in consecutive order of rank**.

Keyword	Rank, median (IQR)	Normalized scale, median (IQR)	Quarters (*N*), out of a possible 34
**VERTIGO SEARCH**			
Vertigo U2	2 (1,3)	90 (60,100)	22
U2	2.5 (2,4)	90 (55,100)	30
Vertigo symptoms	4 (1,10)	65 (20,100)	29
Positional vertigo	4 (3,5)	58 (35,75)	34
Benign vertigo	6 (4,8)	50 (35,70)	34
Vertigo lyrics	7 (4,13)	35 (30,50)	19
Vertigo causes	9 (2,14)	45 (15,75)	31
Benign positional vertigo	9 (6,12)	45 (25,60)	34
Dizziness	10 (8,13)	35 (20,45)	34

*Data from Insights for Search per quarter from 2004 to 2012. “Top searches” are defined as the most common keywords that were searched by users either before or after the keyword of interest (in this case, “vertigo”) was searched*.

**Figure 1:**
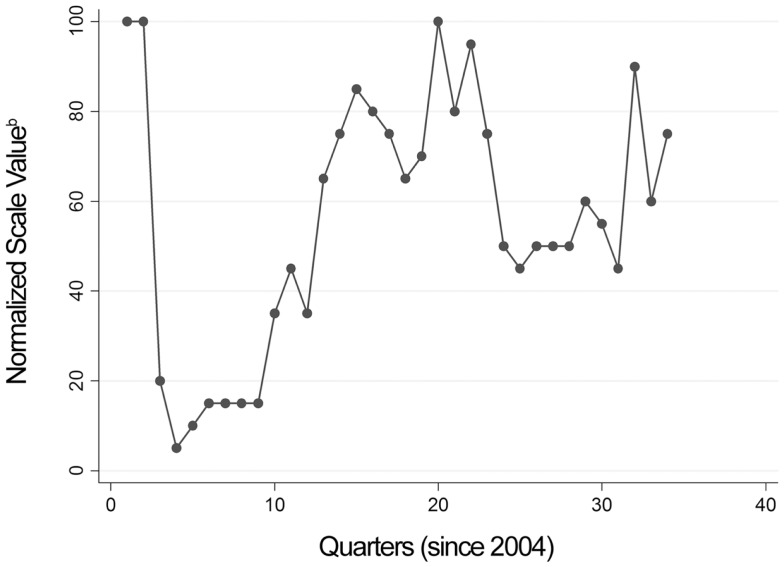
**Scatter plot of the highest normalized scale value for a benign paroxysmal positional vertigo (BPPV) term (any of “positional vertigo,” “benign vertigo,” and “benign positional vertigo”) obtained per quarter since 2004 from *Google Insights for Search***. Note that the drop in normalized scale value from quarter 2 to 3 corresponds to the same time period that the keyword “vertigo U2” became the highest ranked “top search” related to vertigo. Normalized scale value, provided by Google Insights for Search, is calculated by dividing the volume of the keyword by the volume of the highest term during each quarter, and then multiplying by 100.

Many of the terms identified by *Google AdWords* and *Google Insights for Search* were relevant to BPPV (Tables 2 and 3). Four of the BPPV terms (i.e., “positional vertigo,” “benign vertigo,” “benign positional vertigo,” and “BPV vertigo”) had more than 100,000 searches per month. Three of the BPPV terms (i.e., “positional vertigo,” “benign vertigo,” and “benign positional vertigo”) have been in the top searches list related to vertigo searches in each of the 34 quarters from 2004 to 2012 (Table 3). Figure 1 displays the highest normalized scale value for any of these three BPPV terms in each quarter since 2004.

## Discussion

In this study, we aimed to describe internet searches on dizziness as a method to understand consumer demand for this information. We found that generic dizziness keywords are commonly used search terms on Google, with three of the five terms having global searches of more than 1 million per month and US searches over 500,000 per month. Although the purpose behind the searches with the generic dizziness terms cannot be determined with certainty, many of the additional terms that were provided by Google (either as “keyword ideas” or “top searches” related to the dizziness terms) suggested large volumes of searches for medical information about dizziness. The associated volumes for the various BPPV-specific search terms indicate substantial demand via the internet for information about this particular disorder. This demand is not a transient phenomenon. For eight consecutive years of data, BPPV terms have consistently been in the top searches related to vertigo.

The large volume of dizziness searches raises the possibility that the internet could be an important platform for delivering information-based interventions specifically for dizziness. Any such interventions would need to be developed, validated, and tested. Developed internet-based interventions could serve as a resource for patients and providers. In fact, patients and providers are already posting and using BPPV treatment videos on YouTube (6, 23), which is likely in part due to the underuse of evidence-based BPPV management in routine care (11–15) and the conduciveness of BPPV management dissemination via videos.

Effective internet-based tools have been developed for many different health topics, including mental health, addiction, and chronic disease management (8). Guides for development have also been published in an effort to ensure that such tools are safe, user-friendly, and effective (8, 24). We are not aware of any development or testing research regarding internet interventions for BPPV or other dizziness topics, even though BPPV treatment videos are readily available on YouTube (6). Also concerning is the finding that one-third of YouTube BPPV videos have important inaccuracies and that patients are posting comments reporting uncertainty about their diagnosis and other questions about the test and treatment (6). Without adequate development and testing research, there is potential for reduced efficacy or even harms (e.g., inappropriate use leading to worsening symptoms or delays in care) that outweigh benefits.

If effective tools are deployed via the internet, an additional challenge will be attracting target users to the website. Effective advertising campaigns require knowledge of keyword searches by the target population (25), which in this case would be individuals seeking dizziness information for medical reasons. Based on the current findings, an advertising campaign that used the keywords “dizzy” and “vertigo” would likely be very inefficient. Conversely, the use of “positional vertigo,” “benign vertigo,” and “benign positional vertigo” would be particularly suitable for a BPPV website given their likely association with searches regarding BPPV, high volumes of associated searches, and stability in frequency of use over time.

This study has important limitations. The data from this study were only from Google and limited to only English language keywords. Data from other search engines were not available for this study, and thus our estimates of the volume of search for dizziness terms should be considered very conservative. The actual intent of the users entering keyword searches cannot be determined. We do not know the specific formulas that Google uses to generate “keyword ideas” or “top searches” because these are proprietary algorithms. However, we did find that the results remained stable across searches performed at different times and on different computers. The available data does not include any detailed information about the internet users, the number of searches per user, or the website hits that result from these keyword searches. This study does not address important questions regarding internet interventions related to dizziness such as the accuracy or usefulness of the content of currently available websites.

Internet-based tools have the potential to reach large numbers of people who are searching for dizziness information. Additional questions remain such as: What is the content that those initiating searches with dizziness terms are seeking? Is it feasible to perform prospective studies with the population of people who are seeking dizziness information from the internet? Internet clinical tools could then be specifically designed and tested for the target audiences. If dizziness clinical tools (e.g., diagnostic and treatment tools), particularly pertaining to BPPV, were developed, it is likely that they would have a substantial user base. Such interventions could contribute to healthcare efficiencies and patient outcomes.

## Conflict of Interest Statement

The authors declare that the research was conducted in the absence of any commercial or financial relationships that could be construed as a potential conflict of interest.

## Supplementary Material

The Supplementary Material for this article can be found online at http://www.frontiersin.org/journal/10.3389/fneur.2014.00050/abstract
