# Supplementary material for: Consumer Demand for Online Dizziness Information: If You Build it, They may Come
Source: Front Neurol. 2014 Apr 16;5:50. doi: 10.3389/fneur.2014.00050 (PMC3997034; doi:10.3389/fneur.2014.00050)
Supplement: Supplementary file 1 [file DataSheet1.DOCX]

Supplemental Table. Top searches related to generic dizziness search terms presented in consecutive order of rank. Data from Insights for Search, per quarter from 2004-2012. “Top searches” are defined as the most common keywords that were searched by users either before or after the keyword of interest (in this case, a generic dizziness term) was searched.

| Keyword | Rank, Median  (IQR) | Normalized scale, median (IQR) | Quarters (N), out of a possible 34 |
| --- | --- | --- | --- |
| **Dizzy search** |  |  |  |
| Dizzy Gillespie | 2 (1,3) | 95 (70,100) | 33 |
| Dizzy spells | 3 (2,4) | 65 (50,75) | 33 |
| Dizzy lyrics | 4 (3,11) | 50 (33, 60) | 28 |
| Dizzy rascal | 5 (1,13) | 45 (25,100) | 34 |
| Dizzy symptoms | 6 (5,6) | 55 (45,65) | 26 |
| Feel dizzy | 6.5 (3,9) | 55 (40,90) | 22 |
| Get dizzy | 7 (5, 11) | 50 (45,75) | 16 |
| Dizziness | 8 (8,10) | 40 (30,50) | 29 |
| Dizzy head | 10 (9,11) | 35 (35,45) | 22 |
|  |  |  |  |
| **Dizziness** |  |  |  |
| Dizziness symptoms | 1 (1,1) | 100 (100,100) | 17 |
| Symptoms dizziness | 1 (1,2) | 100 (100,100) | 17 |
| Dizziness causes | 2 (2,2) | 78 (58,95) | 28 |
| Nausea | 4 (4,6) | 45 (40,55) | 24 |
| Dizziness pregnancy | 5 (4,6) | 40 (35,45) | 18 |
| Vertigo dizziness | 6 (4,7) | 40 (30, 45) | 18 |
| Vertigo | 7 (3,10) | 40 (35, 45) | 32 |
| Symptoms of dizziness | 7 (5, 9) | 40 (35,40) | 29 |
| Causes of dizziness | 8 (6, 9) | 35 (35, 40) | 28 |
| Dizzy | 10 (8,11) | 35 (30, 35) | 29 |
|  |  |  |  |
| **Lightheaded** |  |  |  |
| Lightheaded wow | 1 (1,2) | 100 (95,100) | 13 |
| Lightheaded addon | 2 (2,4) | 50 (45,65) | 12 |
| Feeling lightheaded | 4 (2,5) | 55 (23,73) | 16 |
| Light headed | 4 (4,5) | 40 (28,58) | 12 |
|  |  |  |  |
| **Lightheadedness** |  |  |  |
| Lightheadedness causes | 1 (1,2) | 100 (85,100) | 17 |
| Dizziness lightheadedness | 2 (2,3) | 85 (80,100) | 11 |
| Dizziness | 2 (1,3) | 95 (83,100) | 16 |
| Lightheadedness and dizziness | 4 (4,5) | 53 (43,73) | 8 |
| Causes of lightheadedness | 6 (5,7) | 40 (40,45) | 13 |
| Symptoms of lightheadedness | 7 (6,8) | 40 (33,40) | 12 |
| Light headed | 10 (9,11) | 30 (25,33) | 8 |
| Dizziness causes | 10 (7,12) | 25 (20,45) | 9 |
